# A Longitudinal Study of Cattle Productivity in Intensive Dairy Farms in Central Ethiopia

**DOI:** 10.3389/fvets.2021.698760

**Published:** 2021-08-12

**Authors:** Rea Tschopp, Gizachew Gemechu, James L. N. Wood

**Affiliations:** ^1^Department of Epidemiology and Public Health, Swiss Tropical and Public Health Institute, Basel, Switzerland; ^2^University of Basel, Basel, Switzerland; ^3^One-Health Unit, Armauer Hansen Research Institute, Addis Ababa, Ethiopia; ^4^Disease Dynamics Unit, Department of Veterinary Medicine, University of Cambridge, Cambridge, United Kingdom

**Keywords:** intensive dairy, Ethiopia, productivity, fertility, mortality, morbidity

## Abstract

Ethiopia is witnessing an emergence of intensive urban dairy farming. The aims of this study were to capture cattle productivity parameters in selected intensive dairy farms in and around Addis Ababa (Central Ethiopia). The study is a pre-requisite and baseline for further economic analysis of diseases such as bovine tuberculosis (BTB) and to assess some of the current challenges faced by farm owners for optimal animal performances. Hence, a 3-year longitudinal observational study was conducted for the first time in Ethiopia, in 24 dairy farms with intensive husbandry, including a total of 1,705 dairy animals. Herd characteristics, animal movement, and productivity parameters (fertility, morbidity, mortality) were recorded in a herd-book. Whereas, half the farms saw their animals increase in number over the 3 years, 37.5% (mainly large farms) saw their herd size decrease. Offtakes accounted for 76.6% of all animal exits. One hundred and ninety (11.1%) animals died of natural causes. Highest mortality was observed in young stock (13.9%). Overall, diseases were the leading cause for death (57.5%). The majority of calves (69%) that died, did so within the first week of life. Mean calving interval (CI) was 483.2 days. Successful conception after artificial insemination (AI) was 66.1% with Addis Ababa and smaller farms faring worst. Mean time interval from calving to first service was 152 days. Date of birth to first service was 592.2 days and date of birth to first calving was 794.7 days. In conclusion, the study showed sub-optimal productivity performances in intensive dairy cattle and highlighted some of the current gaps and challenges in urban dairy productivity.

## Introduction

Ethiopia has the largest livestock population in Africa, with an estimated 60 million cattle (1). The great majority of cattle are local zebus while upgraded cattle (exotic breeds and crossbreeds) account for 0.22 and 1.54%, respectively (1). Ethiopia, thanks to its temperate climate, holds large potential for dairy development. Numerous program and policy interventions were implemented in the last decades to develop the dairy sector. As a result the milk production increased steadily from about 927 million liters in 1996 to 3.3 billion liters in 2018 (1, 2).

The Ethiopian Government has promoted a national development strategy of agricultural-led industrialization encouraging the private sector to get more involved in dairy farming (3). This resulted in the emergence of private intensive dairy farms of various sizes run as a business, which focused on peri-urban and urban areas in the central highlands, taking advantage of urbanized large markets and the rapidly increasing demand for animal products such as milk (4, 5). Traditionally mainly located in and around Addis Ababa, intensive dairy farms are currently emerging in various locations in the Ethiopian Highlands (6, 7). Intensive commercial dairy farming is based on upgraded cattle breed, mainly Holstein-Friesian × Zebu, improved services and purchased conserved feed (8, 9). Almost all of the fluid milk supplied to major urban and peri-urban centers in Ethiopia, comes from these urban and peri-urban smallholder and commercial dairy producers (10, 11).

Despite the overall increased milk production, average milk yield per dairy cow remains low (1). In addition, fertility issues have been highlighted as a major problem in both local and crossbreed cattle in Ethiopia (12–14). Several factors contribute to this low productivity; among them diseases, nutrition, poor management, lack of infrastructure, and veterinary service provision (15, 16).

Longitudinal productivity studies are valuable tools to assess herd productivity, carry out cost-benefit analysis, evaluate potential health, and/or economic impacts of diseases and assess efficacy of interventions. These kind of studies provide a more accurate picture than rapid appraisals or cross-sectional studies. A longitudinal study on productivity of cattle held under traditional husbandry systems was performed in Ethiopia (17), which described herd dynamics, productivity parameters, and main constraints to better productivity. No such studies have so far been performed in the context of the Ethiopian urban dairy industry. Numerous research on dairy cattle productivity were done in Ethiopia but these either included small holders in peri-urban and rural areas, or focused only on a specific productivity parameter or are very much outdated (13, 14, 18, 19).

Current information on productivity in dairy cattle kept under intensive dairy systems in urban areas is lacking. The aims of this longitudinal study were to capture productivity parameters over a period of 3 years in selected intensive dairy farms in and around Addis Ababa (Central Ethiopia) and to assess some of the current challenges faced by farm owners for optimal animal performances.

## Materials and Methods

### Study Sites

This study was carried out in Central Ethiopia within the greater milk production belt, namely Addis Ababa (Kaliti, Kolfe, Yeka, Gulele districts), as well as Debre Zeit (also known as Bishoftu) and Sendafa, 50 and 40 km away from the capital, respectively. Data were collected between November 2015 and September 2018. The area is conducive to dairy farming and has high production potential. It benefits from a temperate climate, abundant rainfall (1,000–1,900 mm/year) with good animal fodder potential and holds the largest high yielding dairy cow numbers in the country (1). The milk shed of the study areas has access to big markets including Addis Ababa (20).

### Study Farms and Animals

This study was part of a larger study assessing Bovine tuberculosis (BTB) in dairy cattle in Central Ethiopia. A list of farms was obtained by district veterinary officers. For our study though, intensive urban dairy farms were purposively selected based on the willingness of the owners to collaborate on a 3-year longitudinal study. These owners were involved in the overall BTB testing program. Budget and logistics allowed for the selection of maximum 30 farms. Purposive sampling can lead to some degree of selection bias. Analysis of herd structure of the overall project in the area and of these selected farms were however very similar, hence giving evidence of representativity. Animals were high milk yielding Holstein-Friesian crossbreed cattle. Farm sizes were represented, and categorized into small farms (3–19 animals), medium farms (20–49 animals), and large farms (50 and more animals). Except for one government farm, all farms were privately owned. Husbandry was similar within farm size categories. Data from farms leaving the study for any reason before year 3 were not taken into account in the final data analysis.

Animals were categorized into sex and age class. Young stock were animals younger than 12 months, replacement stock were animals from 12 months to around 3 years, and breeders from 3 years onwards.

### Tools and Parameters Recorded

An initial registration of all animals was performed in each farm at the start of the study. A herd-book was prepared capturing parameters such as any new animal entry (purchase, birth, gift), animal exit (selling, slaughtering, death), detailed data related to selling and purchasing of animals (e.g., cost, location, reason), morbidities including mastitis, mortalities, and fertility (AI dates, calving dates). The farms were visited twice a month by the same investigators. Herd-book information were updated on hard copies during each visit, and data entered in a Microsoft Access table. General observations were made on-farm during each visit (e.g., husbandry, fodder, discussion with farmers about any problems encountered). All animals were dewormed once a year with Albendazole by the farmers.

During the bi-monthly visits, the investigators who were all trained veterinarians offered advise on diverse husbandry issues and disease management, and provided training on particular topics on those farms that requested it (e.g., better heat detection, husbandry and fodder improvement, calf mortality, diseases).

### Data Analysis

Data were entered into Microsoft Access, from which smaller subsets of data were transferred into Microsoft Excel tables to analyse particular parameters as needed. All data were analyzed using Stata 15 (StataCorp, Texas, USA). Data were analyzed using descriptive statistics. One-way analysis of variance (ANOVA) was used to analyze fertility parameters and results showed as Standard Error (SE), Standard Deviation (SD), 95%CI, and *p*-value. Calving rates, mortality and morbidity rates, offtake rates, and sales rates were derived from the collected data. Offtake rates were the proportion of sold or slaughtered animals in one particular year. Net offtake rates were calculated as the number of animals that were removed from the herd (slaughter/sold) minus the number of animals that were brought into the herd (purchase/gift) divided by the initial herd size (opening number) multiplied by 100.

### Ethical Clearance

This research study was approved by the Institutional Review Board (IRB) of Aklilu Lemma Institute of Pathobiology, Addis Ababa University (reference number IRB/ALIPB/2018), the Institutional Review Board of AHRI (AAERC) (reference number PO46/14), and supported by the Ethiopian Ministry of Livestock and Fisheries.

## Results

### Herd Structure and Dynamic

Out of the 30 farms originally included in the study, six farms left either due to unwillingness to continue the study (*N* = 2), difficulty maintaining a herd-book follow-up (high staff rotation; poor data availability/accuracy) (*N* = 3) or due to farm closure (*N* = 1). Data were collected from 24 dairy farms from November 2015 to September 2018 and included in the final data analysis. Eight farms were categorized as small with a mean herd size of 13.2 animals (95%CI: 10.3–16.1) at initial registration. Eleven farms were medium sized with a mean herd size of 31 animals (95%CI: 24.1–37.9). Five were large farms with a mean herd size of 93.8 animals (95%CI: 65.5–122). A cumulative total of 1,705 animals were included in the study. The overall cumulative herd structure over the entire study duration is shown in Table 1. Female breeders accounted for 40.6% of all females. Of these, 10.9% were older than 10 years. Herd structure was similar in all three farm categories.

**Table 1:** Overall cumulative herd structure categorized by sex and age class.

**Overall herd structure**	**Female**	**Male**	**Total**
Young stock (<1 year)	481 (36)	333 (90.5)	814 (47.7)
Replacement (1–3 years)	313 (23.4)	23 (6.2)	336 (19.7)
Breeder (>3 years)	543 (40.6)	12 (3.3)	555 (32.6)
Total	1,337	368	1,705

Overall, 789 new animals entered the study during the 3 years. The majority were calves born on farms (724; 91.8%), whereas 49 (6.2%) animals were purchased and 16 (2%) were either shared between farms or given as gifts. Females represented 57.5% of all newborn calves. Heifers (*N* = 27) and cows (*N* = 13) represented the majority (81.6%) of all purchased animals.

Overall, 830 animals left the study herds. The majority of exit were due to offtakes (*N* = 638; 76.6%) followed by natural death (*N* = 190; 23%), whereas 3 (0.4%) animals were lost through divorce asset sharing and 1 (0.1%) animal was stolen. Half of all exited animals (offtake and death) were calves (*N* = 417; 50.2%), a third were adult breeders (*N* = 281; 33.9%), and the others replacements (*N* = 132; 15.9%).

Half of the farms (50%) saw their animal numbers increase over the 3 years (total herd increase by 42%), 3 (12.5%) remained stable whereas 9 farms (37.5%) saw their animal numbers decrease (total herd decrease by 30.6%), including 4 out of the 5 large farms (80%). Geographical location (*p* = 0.33) and farm size (*p* = 0.163) were not statistically associated with change in herd size.

### Offtakes and Natural Death

During the study, 190 (11.1%) animals died of natural causes. Table 2 shows the mortality and offtakes by age and sex during the entire study.

**Table 2:** Mortality and offtake numbers by age and sex during the entire study period.

		**Natural death**		**Offtakes**	
**Age and sex category**	**Total cumulative numbers**	**Total animal number**	**% of category**	**Total animal number**	**% of category**
Young stock	814	113	13.9	263	32.3
*Female*	*481*	*63*	*13.1*	*24*	*4.9*
*Male*	*333*	*50*	*15*	*239*	*71.7*
Replacement	273	17	6.2	53	19.4
*Female*	*238*	*15*	*6.3*	*38*	*15.9*
*Male*	*35*	*2*	*5.7*	*15*	*42.8*
Breeder	657	60	9.1	323	49.1
*Female*	*645*	*60*	*9.3*	*320*	*49.6*
*Male*	*12*	*0*	*0*	*3*	*25*
Total	1705	190	11.1	639	37.4

The highest mortality was observed in young stock (13.9%), where mortality in males (15%) was higher than in females (13.1%).

**Figure 1:**
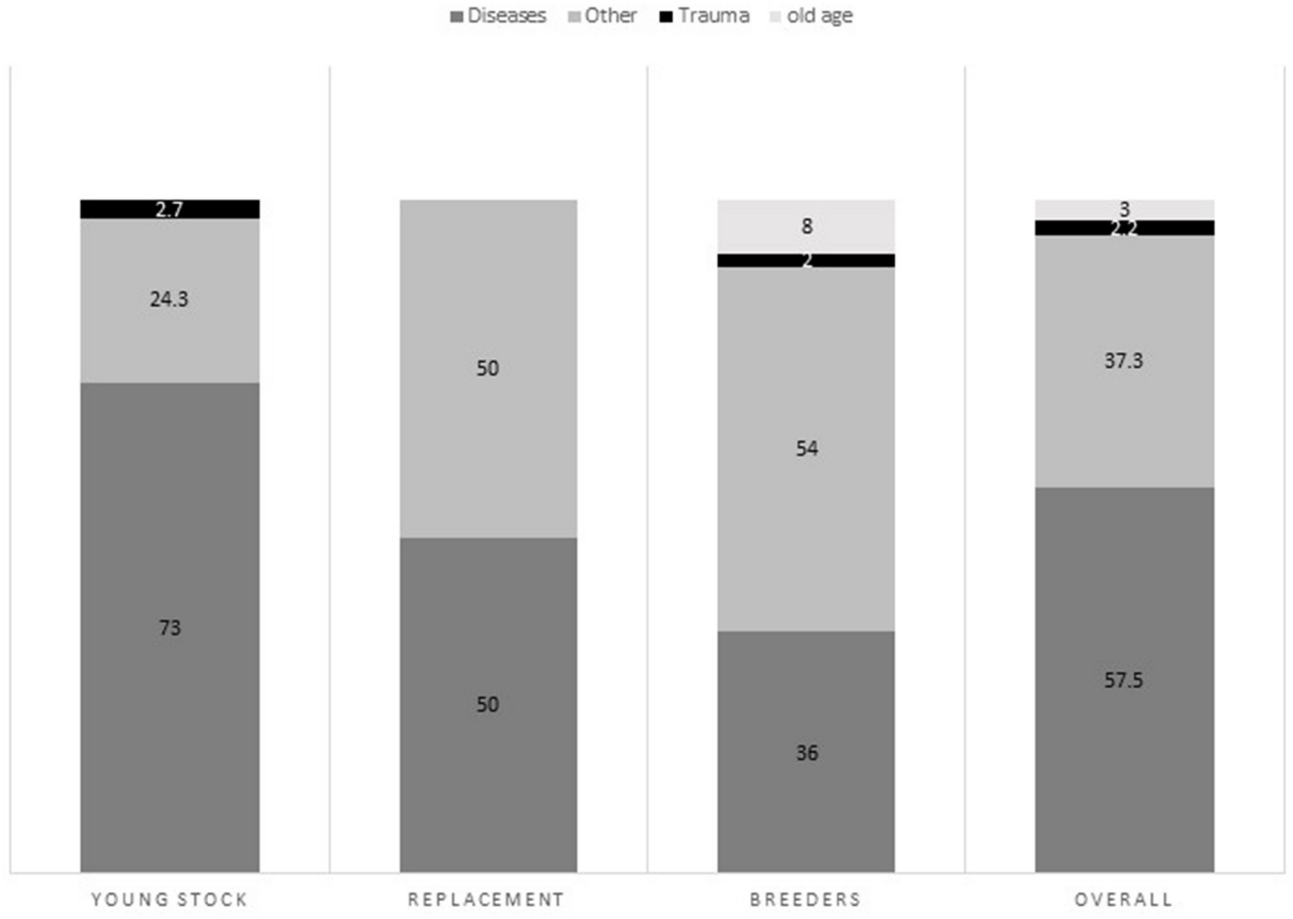
Overall cause of death (categorized) as reported by the farmers (combining age and sex).

The cause of death was known for 134 animals (see Figure 1). Overall, infectious diseases were the leading cause of animal mortality.

Among the 39 calves for which detailed information on the cause of death existed, besides infectious diseases, diarrhea accounted for the highest mortality (*N* = 17; 43.6%). Unexplained progressive weakness, emaciation, and refusal to drink caused death in 10 calves (25.7%), 5 (12.8%) died at birth, 4 (10.2%) animals died of respiratory distress, 2 of bone deformation/lameness, 1 had swallowed a foreign body and choked, another 3 died suddenly.

The majority (*N* = 78; 69%) of all calves died during their first week of life, whereas 87.6% (*N* = 99) died before the age of 6 months.

Among breeders, a third (34%) of the deaths were caused by digestive problems including metabolic problems, 25% were caused by parturition related problems and another 25% by infectious diseases [rabies, Foot- and Mouth Disease (FMD), and Bovine Viral Diarrhea (BVD)]. Hypocalcaemia (milk fever) was the cause of 13.6% of all female breeder's death. Another 13.6% died after experiencing progressive emaciation and weakness.

Table 3 shows the crude offtake rates (COR) and the net offtake rates (NOR) per age group and sex for year 1 and year 2. Year 3 was an incomplete 12 months period and hence data were not used in this table. The biggest offtake rates were observed in male calves (NOR = 70.6%). The majority of male calf offtakes happened in the first month of life (81.6%). A 185 (77.4%) calves were removed from the herd between the age of one and 15 days. Nineteen (79%) female calves were removed when aged 5 months or older.

**Table 3:** Crude offtake rates (COR) and Net offtake rates (NOR) by age and gender for the first and second year of the study.

	**Number animals**		**Number offtakes**		**Number purchases**		**COR (%)**		**NOR (%)**			
**Age**	**Year 1**	**Year 2**	**Year 1**	**Year 2**	**Year 1**	**Year 2**	**Year 1**	**Year 2**	**Year 1**	**Year 2**	**Average COR (%)**	**Average NOR (%)**
Young stock	365	457	137	115	0	2	37.5	25.1	37.5	24.7	31.3	31.1
Female	230	316	38	18	0	1	16.5	5.7	16.5	5.4	11.1	10.9
Male	135	141	99	97	0	1	73.3	68.8	73.3	68	71	70.6
Replacement	308	273	112	71	11	21	36.4	26	32.8	18.3	31.2	25.5
Female	289	255	110	71	9	20	38	27.8	35	20	32.9	27.5
Male	19	18	2	0	2	1	10.5	0	0		5.2	0
Breeder	538	433	240	143	49	48	44.6	33	35.5	21.9	38.8	28.7
Female	528	422	219	140	47	45	41.5	33.2	32.6	22.5	37.3	27.5
Male	10	11	3	3	2	3	30	27.3	10	0	28.6	5
Total	1,211	1,163	471	329	60	71	38.9	28.3	33.9	22.2	33.6	28

### Morbidity

Overall, 297 animals were reported sick during the study. Infectious diseases (33.7%), leg problems (lameness, arthritis, wounds, edema, abscess) (19.9%), and diarrhea (13.1%) were the three most recorded problem categories, followed by fertility/genital tract related problems (7.4%), respiratory (2.7%), and others (23.2%).

**Figure 2:**
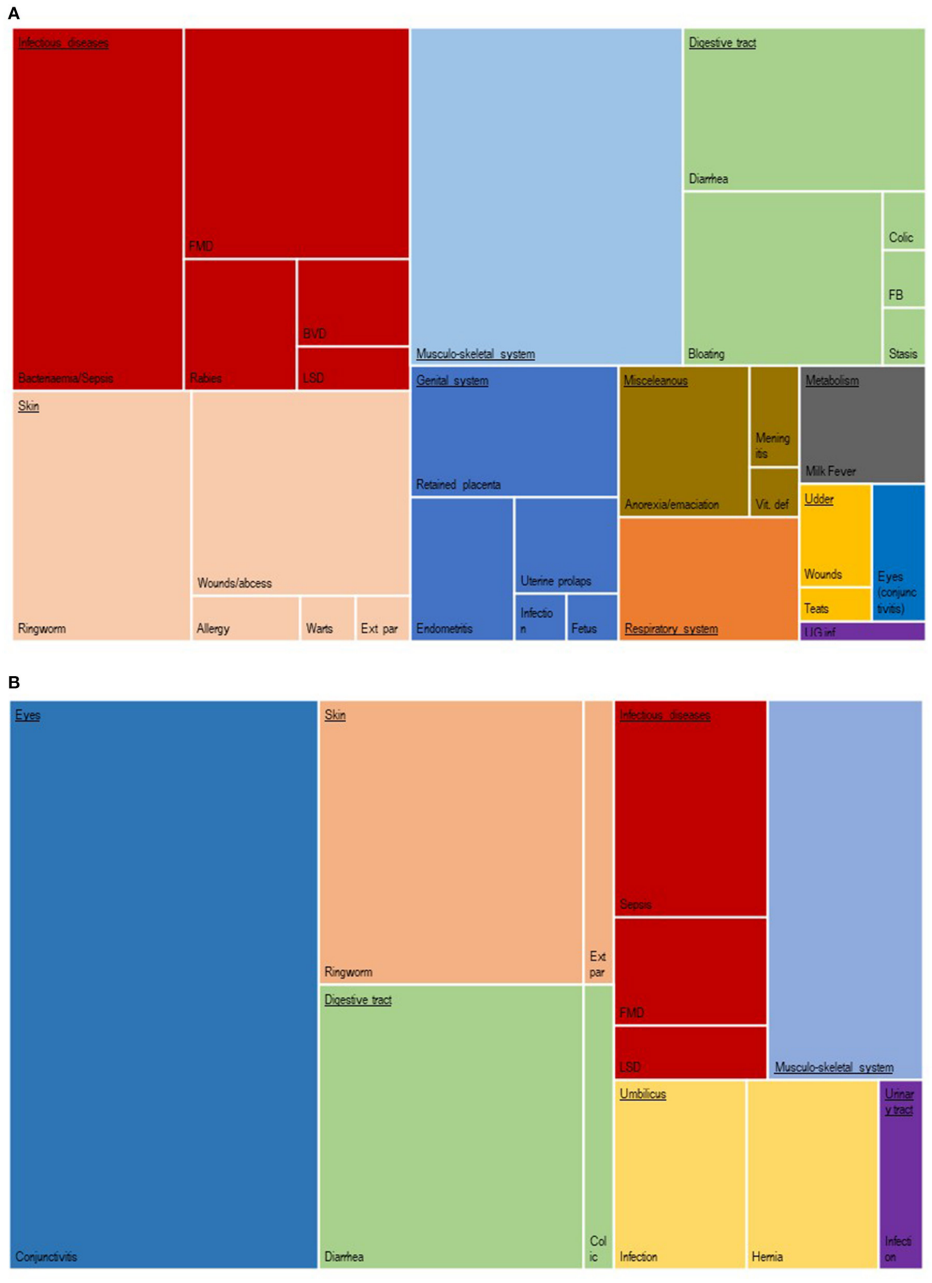
Detailed health problems as recorded in 288 animals (**A** in 225 replacement/breeder animals; **B** in 63 young stock).

Detailed information on morbidity was recorded in 225 replacement/breeders and 63 young stock (see Figures 2A,B).

In replacements and breeders, the leading causes of morbidity were known infectious diseases (*N* = 58; 25.8%) such as BVD, FMD, Lumpy skin disease (LSD), and rabies. Skin diseases ranked second with 40 cases (17.7%), followed by musculoskeletal problems (16.4%).

In addition, clinical mastitis was recorded in 76 animals (heifers and breeders), which accounts for approximately 9% morbidity. This number is likely severely underestimating the true mastitis incidence (personal observation).

### Fertility

Table 4 shows the results for four fertility parameters for each farm size category. Generally, larger farms tended to show better performances with a clear statistical difference (*p* = 0.007) for calving to next service interval as compared with the other farms.

**Table 4:** Fertility parameters during the study period.

**Parameter**	**Farm size**	**Total number**	**Mean days**	**SE**	**CI for mean (days)**	**Min–Max (days)**	***P*-value**
DOB to first calving	Small	6	785	37.9	687.7–882.3	691–950	0.125
	Medium	14	740.1	32.1	670.7–809.5	617–1,044	
	Large	19	837.9	41.3	751–924.8	455–1,144	
	*Overall*	*39*	*794.7*	*24.5*	*744.9–844.4*	*455–1,144*	
DOB to first service	Small	10	592.2	14.7	563.1–621.4	422–675	*0.175*
	Medium	23	522.4	25.1	470.4–574.4	380–874	
	Large	53	642.9	17.4	607.9–678	428–967	
	*Overall*	*86*	*592.2*	*14.7*	*563.1–621.4*	*380–967*	
Calving to next service	Small	54	168.5	11.7	144.9–192	55–436	*0.007*
	Medium	84	158	11.8	134.6–181.5	21–669	
	Large	255	146.6	5.1	136.5–156.8	4–659	
	*Overall*	*393*	*152*	*4.5*	*143.2–160.9*	*4–669*	
Calving interval	Small	24	445.3	24.9	394.5–496.2	282–849	0.141
	Medium	67	462.5	15.3	431.9–493.2	292–967	
	Large	197	494.8	13.1	494.1–546	272–941	
	*overall*	*288*	*483.2*	*8.4*	*466.7*–*499.7*	*272–967*	

*DOB, date of birth*.

Forty-six animals out of 870 animals that had received at least one artificial insemination (AI) had aborted (5.3%).

Out of 610 animals that received AI, 341 had calved successfully (55.9%), whereas 175 failed to calve after 9 months (28.7%). Ninety-four were slaughtered or sold before the end of the 9 month pregnancy period (15.4%).

Overall, one-third of the AI did not lead to a successful calving. The conception rate decreased as the farm size decreased, and was lower in Addis Ababa as compared to the other geographical locations (see Table 5).

**Table 5:** Number of successful AIs by farm size category and geographical locations.

**Category**		**Total AI**	**Total failed conception after AI (%)**	**Total successful conception after AI (%)**	**Anova Bartlett's test *p*-value**
Farm size	Small (3–19)	56	28 (50)	28 (50)	0.213
	Medium (20–49)	147	62 (42.2)	85 (57.8)	
	Large (50+)	313	85 (27.1)	228 (72.8)	
Location	Addis	257	112 (43.6)	145 (56.4)	0.06
	Debre Zeit	136	35 (25.7)	101 (74.3)	
	Sendafa	123	123 (22.8)	95 (77.2)	
Total		516	175 (33.9)	341 (66.1)	

The numbers of AI provided per animal ranged from one to ten, with a median (50th percentile) of one (Table 6). Indeed, the majority of animals (65.4%) only had one AI; 83.9% received one or two; 14.2% received between three and five; and 1.9% received six or more AIs (Table 6).

**Table 6:** Number of artificial insemination given per cow by farm-size category.

**Number of AI/cow**	**Small farm**	**Medium farm**	**Large farm**	**Total**
1	57 (58.8)	163 (72.8)	337 (63.5)	557 (65.4)
2	19 (19.6)	38 (17)	101 (19)	158 (18.5)
3–5	17 (17.5)	22 (9.8)	82 (15.4)	121 (14.2)
≥6	4 (4.1)	1 (0.4)	11 (2)	16 (1.9)
Total	97	224	531	852

## Discussion

Knowledge of herd structure, fertility, mortality, and offtake rates are key parameters for determining the population dynamics and herd productivity in the dairy sector at both farm and country level.

Long-term longitudinal studies have pros and cons. There is always the risk of participation bias, drop-outs during the study and delayed data analysis and results. They require a substantial time effort, which is also often linked to logistical and financial challenges and limitations. However, these type of studies produce more accurate and reliable data, reduce recall biases and are closer to the reality of event timelines, allowing to assess cause-effects more accurately. Representativeness of the larger population is always a limitation. In our study, we compared herd structure and farm size with the ones provided by the larger project for Central Ethiopia and they were very similar, thus providing some indication of representativeness. However, results translation to the larger national dairy cattle population should be done with care.

Major challenges faced in our study to record productivity were: lack of animal identification, lack of record keeping, high staff turn-over, lack of interest of owners, and depopulation or closing down of farms. Only a few farms (including all large farms) had their animals officially tagged despite the Ethiopian Government's efforts to implement a livestock identification system using plastic ear tags through the National Artificial Insemination Center (NAIC). The Ethiopian Ministry of Livestock and Fisheries launched a national livestock identification and traceability system (LITS) with the financial and technical support of the U.S. Agency for International Development (USAID) (21). Unfortunately, the system was not fully implemented during the time of our study. Owners would rely on their own identification system (individual name; own ear tag number) that were sometimes replaced entirely after some time with other ear tag numbers. Re-using ID tags of existing animals for a new animal was common. Accurate and updated record keeping of animals and productivity parameters were performed only in two large farms (8.3% of the farms). Two other large farms kept records on animal identification and some basic animal data but rarely updated the information on productivity. Hence the need for the study to introduce an independent animal registration and herd book that could be followed up for 3 years. In addition, dairy farm staff in medium and large farms were often untrained and showed high work turn-over. This likely contributed to poor animal management in many of the dairy farms. Nine farms (37.5%), including 4 out of the 5 large farms, decreased their number of animals. Reasons given included diseases, high running costs, poor availability of fodder, and poor animal productivity.

Dairy farms kept mainly female animals (78.4%). Among the males, the great majority were young calves (90.5%). Male calves represented also the biggest offtake (70.6%). They were largely removed from the farm during the first month of life, with 81.6% removed within the first 14 days of life. Currently not much veal meat is produced; it comes from very young calves (younger than 1 month old) with a market directed mainly to expatriate communities living in the capital city. Offtakes represented the majority of all animal exits, whereas natural death accounted for a quarter of the exits. Farms relied predominantly on own breeding rather than purchasing new animals (*N* = 49; 6.2%). Low numbers of purchased animals are ideal from a biosecurity point of view as the additional risk and burden of importing new diseases into the farm remains low at farm level.

Highest mortality was found among young stock (13.9%), which is higher than calf mortality of 9.3% found in small-holders in Hawassa (22) but in line with results found in calves from dairy farms by Romha et al. (23) in Debre Zeit (Bishoftu). The latter study observed that lack of colostrum provision was the major cause of mortality and diarrhea in calves. In our study, we did not follow up on the provision of colostrum. However, diarrhea accounted for 43.6% of calf mortality suggesting husbandry issues. Diarrhea was also observed by Megersa et al. (22) to be the leading cause of calf morbidity. A quarter of calves (25.7%) died following a period of progressive emaciation and reluctance to take milk, also suggestive of possible husbandry problems. Farmers acknowledged that calf mortality was one of the major issues on their farm in addition to fertility problems. Mortality in male calves (15%) was a slightly higher than in female calves (13.9%). This might reflect the fact that male calves were removed mostly within the first 2 weeks of life and were thus neglected. Death during birth or shortly after birth was recorded in 12.8% of calves. High neonatal calf mortality was also observed in dairy farms by Fentie et al. (24). High calf mortality was one of the major complaints of the farm owners in our study. Among breeders, a quarter (25%) died due to parturition problems (e.g., birthing, endometritis, uterine prolapse). These results highlighted poor obstetric practices and husbandry as well as poor nutritional management (25). A third of all deaths in adults were caused by digestive/metabolic problems hinting again to poor husbandry and feeding practices. Milk fever is a metabolic disease caused by blood calcium deficiency around birth time, leading to poor labor activity (dystocia), birth of weak calves or stillborn, and can be fatal for cows. In our study, 13.6% of breeders who died had milk fever. Gammada (26) also observed 17.3% of milk fever cases in small-scale dairy farms in Jimma. Hypocalcaemia, which was probably under-reported, could also be a cause for the high level of parturition issues and high calf mortality at birth. The condition is more prevalent in pluriparous cows with higher milk production, and is strongly linked to feeding management during the weeks prior to calving. A previous study by GebreMichael et al. (9) showed that 70% of the study farms did not adapt the diet of their pregnant cows during the critical dry period.

Husbandry and fodder issues were reflected in several of the reported animal morbidities. Leg problems involving arthritis, wounds, and abscesses were reported in 19.9% of the animals. In addition, not recorded but observed by the researchers was the high prevalence of untrimmed hooves in all farms, leading to chronic hoof deformities and lameness. Chronic pains and inability to walk, jump, and display normal behavior can have an impact on fertility (poor heat detection) but also on milk productivity (27). The many metabolic issues reported such as bloating and milk fever are indications of inadequate fodder intake and feeding management. Anecdotally, some farmers were observed giving colostrum to drink to cows that had calved, leading to serious bloating. Infectious diseases were the top causes for animal morbidity (33.7%). Infectious diseases, such as Blackleg and LSD were also previously observed being leading causes of morbidities in small-scale dairy farms in Jimma (26). In our study, Besides bacterial septicemia, FMD, LSD, and BVD were recorded. Foot- and mouth disease is an endemic disease in Ethiopia with sero-prevalence in Central Ethiopia ranging between 14.5 and 30% (28–30). Lumpy skin disease prevalence in dairy cattle in Central Ethiopia ranges from 22.5 to 33.9% with mortality up to 7.4% (31, 32). Seropositivity of BVD was reported in dairy cattle as high as 32.6% (33). Infectious diseases can have a range of impacts on animal productivity. In our study, none of the farms had consistent appropriate biosecurity measures in place. The reasons were not specifically investigated as part of the study but included lack of awareness and cost. Infectious diseases were also the leading cause of death. In adults, rabies, FMD and BVD caused 25% of all deaths during the study period. LSD and FMD vaccines are produced in-country, though sometimes with limited capacity. Most farmers do not regularly vaccinate their cattle and rather wait for an outbreak to do so often worsening the outbreak situation and increasing complaints from farmers about the vaccine efficacy (NVI, personal communication).

Mean calving interval (CI) in our study was 483.2 days (16 months). This is a sub-optimal CI as compared to the ideal 365 days for dairy cattle (34). Studies on reproductive performances in intensive dairy cows in Ethiopia are sparse or outdated (35). Twenty years ago, studies done in dairy cattle in Central Ethiopia showed a CI ranging between 435.2 and 544.9 days (average 490 days) (18), which indicates the fertility performances have not much improved. Several factors can contribute to this long CI. Lobago et al. (19) showed that 67.4% of cows showed a delayed return to ovarian cyclicity after calving (longer than 55 days). Prolonged postpartum anestrus is most likely linked to inadequate nutrient intake. Other factors influencing the CI include the ideal timing of post breeding pregnancy testing (36) and proper service. In our study the majority of cows (65.4%) received only one AI. Besides financial constraints, this was mostly due to the lack of qualified insemination technicians or technicians not coming to the farm after the call and thus missing the estrus period. On the other hand, some animals in large farms received up to 10 AIs. Large farms sometimes have assigned AI technicians who are on call. The mean time between calving and the first AI given was 152 days (CI: 143.2–160.9). This was longer than the reported 115 days by Tadesse et al. (35) and much longer than the ideal calving-first insemination interval. Intervals between two AIs without a calf being produced ranged between 1 and 408 days. The median interval was 59.5 days (CI: 49.1–66 days). Animals were sometimes inseminated although not showing signs of estrus, e.g., during the first couple of weeks after calving (7.2% of AIs), during pregnancy, or the AI technician inseminated several animals the same day to reduce the numbers of visits even if outside the possible estrus period. 40.5% of AIs were repeated after 2.5 months. Poor estrus expression, detection by the owner, and poor AI techniques (timing, conception rate, pregnancy diagnosis, etc.), led to poor service per conception outputs. Infectious diseases are also known to affect fertility and cause diverse reproductive disorders. In intensive dairy cattle in central Ethiopia, high prevalence of *Neospora caninum* and Schmallenberg virus were both observed (37–39), whereas BVD was linked in Jimma (Western Ethiopia) with reproductive disorders in cattle (40).

Overall, 28.7% of all inseminations failed to conceive a calf after 9 months. The conception rate was better the bigger the farm. Small farms fared the worst as well as Addis Ababa (56.4%) as compared to the other sites. Abortion rate was in our study 5.3%. Previously reported abortion rates ranged between 1.7 and 20.2% (18, 41).

Our study showed overall sub-optimal productivity performances in intensive dairy farms, likely contributing to substantial financial losses at farm level. Keeping accurate herd book follow-up data would help identifying correctly and swiftly animals with reproductive problems, hence would help improve farm productivity. Record keeping helps managing the question of profitability to continue to inseminate animals with problems and this will increase farm profit. The problems highlighted in this study are complex and often interlinked. A holistic approach is needed in order to improve overall animal productivity. Poor husbandry was observed to be a major contributor to poor productivity, affecting among others calf mortality, and animal fertility. These were also the two major topics that farmers would regularly raise during our entire study and questioning how to address them.

## Conclusion

Longitudinal productivity studies and herd-book follow-ups are great tools to identify productivity challenges and establish baseline productivity data, on which further economic studies can be built upon. Despite the growing importance of the dairy industry in Ethiopia, accurate data on dairy cattle productivity under intensive farming is often lacking. Larger sample size would be warranted to corroborate our findings. Besides improving animal tracking (ear-tagging that is reliable and centralized), keeping accurate animal records and tackling prevalent infectious diseases, emphasis should be urgently given to improving overall animal husbandry and feeding, all of which would ultimately lead to better productivity and better animal welfare. Continuous training opportunities of farmers and their staff is a key step toward improving all the above mentioned aspects. Certifications of farms, and creation of model-farms can further help improve training quality and standard, and provide a positive deviance approach to better animal husbandry.

## The ETHICOBOTS Consortium

The members of the Ethiopia Control of Bovine Tuberculosis Strategies (ETHICOBOTS) consortium are: Abraham Aseffa, Adane Mihret, Bamlak Tessema, Bizuneh Belachew, Eshcolewyene Fekadu, Fantanesh Melese, Gizachew Gemechu, Hawult Taye, Rea Tschopp, Shewit Haile, Sosina Ayalew, Tsegaye Hailu, Armauer Hansen Research Institute, Ethiopia; Rea Tschopp from Swiss Tropical and Public Health Institute, Switzerland; Adam Bekele, Chilot Yirga, Mulualem Ambaw, Tadele Mamo, Tesfaye Solomon, Ethiopian Institute of Agricultural Research, Ethiopia; Tilaye Teklewold from Amhara Regional Agricultural Research Institute, Ethiopia; Solomon Gebre, Getachew Gari, Mesfin Sahle, Abde Aliy, Abebe Olani, Asegedech Sirak, Gizat Almaw, Getnet Mekonnen, Mekdes Tamiru, Sintayehu Guta, National Animal Health Diagnostic and Investigation Center, Ethiopia; James Wood, Andrew Conlan, Alan Clarke, Cambridge University, United Kingdom; Henrietta L. Moore and Catherine Hodge, both from University College London, United Kingdom; Constance Smith at University of Manchester, United Kingdom; R. Glyn Hewinson, Stefan Berg, Martin Vordermeier, Javier Nunez-Garcia, Animal and Plant Health Agency, United Kingdom; Gobena Ameni, Berecha Bayissa, Aboma Zewude, Adane Worku, Lemma Terfassa, Mahlet Chanyalew, Temesgen Mohammed, Miserach Zeleke, Addis ababa University, Ethiopia.

## Data Availability Statement

The datasets presented in this article are not readily available because Project regulations of the Ethicobots consortium project (PI: JW). Requests to access the datasets should be directed to JW (jlnw2@cam.ac.uk).

## Ethics Statement

This research study was approved by the Institutional Review Board (IRB) of Aklilu Lemma Institute of Pathobiology, Addis Ababa University (reference number IRB/ALIPB/2018), the Institutional Review Board of AHRI (AAERC) (reference number PO46/14), and supported by the Ethiopian Ministry of Livestock and Fisheries. Written informed consent for participation was not obtained from the owners because a verbal informed consent was provided by the participants.

## Author Contributions

RT has designed the research, performed the data analysis, and drafted the manuscript. GG has collected field data and contributed to drafting the manuscript. JW provided critical comments to the Manuscript. All authors have contributed to the manuscript, and have approved the submitted version.

## Conflict of Interest

The authors declare that the research was conducted in the absence of any commercial or financial relationships that could be construed as a potential conflict of interest.

## Publisher's Note

All claims expressed in this article are solely those of the authors and do not necessarily represent those of their affiliated organizations, or those of the publisher, the editors and the reviewers. Any product that may be evaluated in this article, or claim that may be made by its manufacturer, is not guaranteed or endorsed by the publisher.
